# Acute Phase IL-10 Plasma Concentration Associates with the High Risk Sources of Cardiogenic Stroke

**DOI:** 10.1371/journal.pone.0120910

**Published:** 2015-04-29

**Authors:** Otso Arponen, Antti Muuronen, Mikko Taina, Petri Sipola, Marja Hedman, Pekka Jäkälä, Ritva Vanninen, Kari Pulkki, Pirjo Mustonen

**Affiliations:** 1 Kuopio University Hospital, Diagnostic Imaging Centre, Department of Clinical Radiology, Kuopio, Finland; 2 University of Eastern Finland, Institute of Clinical Medicine, Unit of Radiology, Kuopio, Finland; 3 Heart Center, Kuopio University Hospital, Kuopio, Finland; 4 NeuroCenter, Kuopio University Hospital, Kuopio, Finland, and Unit of Neurology, University of Eastern Finland, Institute of Clinical Medicine, Kuopio, Finland; 5 Department of Clinical Chemistry, University of Eastern Finland, Kuopio, Finland; 6 Eastern Finland Laboratory Centre, Kuopio, Finland; 7 Keski-Suomi Central Hospital, Department of Cardiology, Jyväskylä, Finland; Heart Research Institute, AUSTRALIA

## Abstract

**Background:**

Etiological assessment of stroke is essential for accurate treatment decisions and for secondary prevention of recurrence. There is evidence that interleukin-10 (IL-10) associates with ischemic stroke. The aim of this prospective study was to assess the levels of IL-10 in ischemic stroke with unknown or suspected cardiogenic etiology, and evaluate the correlation between IL-10 plasma concentration and the number of diagnosed high risk sources for cardioembolism.

**Methods:**

A total of 141 patients (97 males; mean age 61±11 years) with acute ischemic stroke with unknown etiology or suspected cardiogenic etiology other than known atrial fibrillation (AF) underwent imaging investigations to assess high risk sources for cardioembolic stroke established by the European Association of Echocardiography (EAE). IL-10 was measured on admission to the hospital and on a three month follow-up visit.

**Results:**

Acute phase IL-10 concentration was higher in patients with EAE high risk sources, and correlated with their number (p<0.01). In patients with no risk sources (n = 104), the mean IL-10 concentration was 2.7±3.1 ng/L (range 0.3–16.3 ng/L), with one risk source (n = 26) 3.7±5.5 ng/L (0.3–23.6 ng/L), with two risk sources (n = 10) 7.0±10.0 ng/L (1.29–34.8 ng/L) and with three risk sources (n = 1) 37.2 ng/L. IL-10 level was not significantly associated with cerebral infarct volume, presence of previous or recent myocardial infarction, carotid/vertebral artery atherosclerosis, paroxysmal AF registered on 24-hour ECG Holter monitoring or given intravenous thrombolytic treatment.

**Conclusion:**

IL-10 plasma concentration correlates independently with the number of EAE cardioembolic risk sources in patients with acute stroke. IL-10 may have potential to improve differential diagnostics of stroke with unknown etiology.

## Introduction

Stroke is the third highest cause of mortality in industrialized countries, accounting for 10% of all deaths, and is the leading cause of long-term disability worldwide [1,2]. The currently recognized mechanisms of ischemic stroke and transient ischemic attack (TIA) are thrombosis, embolism, or decreased perfusion [3]. Brain embolism can be of arterial or cardiac origin, with atrial fibrillation (AF) being by far the most common cause of cardioembolic stroke/TIA [4,5]. Exclusion of hemorrhage and accurate etiological assessment are vital for appropriate treatment and secondary prevention of stroke. Despite current imaging techniques and modern diagnostic work-up, approximately 30−40% of patients with ischemic strokes/TIAs remain without a well-defined etiology [6]. A cardiac origin for ischemic stroke has traditionally been suspected on the basis of characteristic clinical symptoms and, more recently, by imaging findings established by the European Association of Echocardiography (EAE) [7–9].

Interleukin-10 (IL-10) is an anti-inflammatory cytokine [10]. IL-10 concentrations have been found to be elevated in the cerebrospinal fluid (CSF) in patients with acute ischemic stroke [11]. It has been suggested that increased IL-10 concentration has a neuroprotective function [12,13]. High serum concentration of IL-10 predicts the presence of salvageable ischemic tissue after ischemic stroke [14,15]. In rodent models of stroke, systemically administrated IL-10 reduces the cerebral infarction volume after stroke [13]. In addition, the number of peripheral blood mononuclear cells (PBMC) secreting IL-10 has been shown to be elevated in patients with ischemic stroke and cerebral hemorrhage [16]. We prospectively studied patients with acute ischemic stroke with unknown etiology or suspected cardiogenic etiology other than known AF. The purpose of the study was to assess the plasma levels of IL-10, and evaluate whether IL-10 plasma concentration is associated with the number of EAE cardioembolic risk sources [17].

## Materials and Methods

The study was approved by the University Hospital Research Ethics Board. Prior to participation in the study, written informed consent was obtained from the patient or the patient's legally authorized representative.

### Study Design and Population

Patients with acute stroke/TIA admitted to university hospital of Eastern Finland were evaluated as candidates for this EMBODETECT study. The neurologists involved in this study recruited 162 patients with unknown etiology or suspected cardiogenic etiology other than known AF, as described previously [7].

Of the 162 patients initially recruited, 21 were excluded; eight patients lacked the IL-10 analyses in the acute phase, cardiac CT (cCT) image quality was not appropriate for analyses in ten patients due to technical error, and three patients decided not to participate after giving informed consent. Altogether 141 patients underwent IL-10 plasma concentration analyses in the acute phase. All patients were invited to a follow-up visit three months later, but only 69 (49%) turned up. According to medical records none of the patients died within 3 months.

### CT Imaging of the Heart, Carotid/Vertebral Arteries and Brain

Profound imaging investigations including combined examination of the heart, aorta, and cervicocranial arteries with CT (CACC-CT) and echocardiography were performed to define the etiology of stroke according to the the EAE high risk source categories for cardioembolism.

In patients *without* cardioembolic high risk sources, the etiology of stroke was categorized according to the TOAST criteria [9]. The volume of the infarcted cerebral tissue was quantified.

All 141 stroke/TIA patients underwent the contrast-enhanced CT scan (Somatom Sensation 16 and Somatom Definition AS; Siemens Medical Solutions, Forchheim, Germany) of the aortic arch, cervical arteries and intracranial arteries, immediately followed by scanning of the ascending aorta and heart. Cardiac imaging was performed during mid-diastole in all study subjects. For volume measurements of infarcted cerebral tissue, 4.5–5 mm transversal slices were reconstructed in the brain CT in the subacute phase (> two days). Infarction volume was calculated by using Simpson’s method [18]. Carotid and vertebral artery stenoses were evaluated from CT angiography (CTA). TEE and CT were used to diagnose aortic arch atheromas. Atheroma plaques with thickness ≥4 mm, ulceration ≥2 mm or aortic atherosclerosis with intimal thickening <4 mm were regarded as high risk sources.

### Additional Imaging with Echocardiography and Magnetic Resonance Imaging

Transthoracic (TTE) and transesophageal echocardiography (TEE) were performed for all patients (Vivid 7 cardiovascular ultrasound system, GE Medical Systems, Buckinghamshire, UK) by several cardiologists as part of their clinical routine [8]. Contrast-enhanced cardiac magnetic resonance imaging (MRI) was performed according to the protocols of the Society of Cardiovascular Magnetic Resonance using a 1.5 T scanner and 12-element phased-array surface coil (Siemens Avanto, Erlangen, Germany), to confirm suspected structural abnormalities in cCT (n = 18) or in patients with discrepant findings in cCT and echocardiography (n = 12). Ambulatory 24-hour Holter ECG was performed to evaluate the presence of paroxysmal AF for all patients.

### IL-10 measurement

Blood samples for IL-10 analyses were taken 3±2 days after hospitalization (range 0–10 days), representing the acute phase, and also at 3 months, representing the chronic phase, by medical laboratory technologists. Plasma samples were separated with centrifugation and stored frozen at -70°C until analyzed. IL-10 concentrations were measured from EDTA plasma samples with sandwich-type high-sensitivity ELISA (Quantikine, R&D Systems, Minneapolis, MN, USA). The sensitivity of the assays was 0.09 ng/L.

### Statistical Analyses

Continuous variables with normal distribution are presented as mean±SD, and categorical variables as absolute values and percentages. Based on the Kolmogorov-Smirnov test, Student’s t-test for normally distributed and the Mann-Whitney U test for abnormally distributed nonparametric values, these tests were used to compare significance in IL-10 concentration between dichotomous groups.

The Kruskal-Wallis test was used when the difference in continuous variable (IL-10 concentration) was tested with ordinal variable with three or more groups (the number of cardioembolic risk sources). Spearman’s correlation coefficient was used to investigate the associations between continuous background characteristics and absolute IL-10 plasma concentration, and the chi-squared test was used test to investigate nominal variables. The ANCOVA test was used to test the effect of patient characteristics on the correlation between IL-10 concentration and the number of cardioembolic risk sources. Statistical significance was set at p<0.05 and high statistical significance at p<0.01. The Bonferroni correction to control the familywise error rate was used for multiple group comparisons (evaluation of statistical significance in 13 EAE high risk sources) [17]; statistical significance was adjusted to p≤0.003. Data were analyzed using SPSS for Windows (version 19, 1989–2010 SPSS Inc., Chicago, USA).

## Results

### Patient Characteristics and Stroke Etiologies

Patient characteristics, prevalence of common risk factors and different stroke etiologies are presented in Table 1. IL-10 plasma concentrations were evaluated in 141 patients (97 males; mean age 61±11 years; range: 32–84 years) in the acute phase and in 69 patients (49 males; mean age 60±11 years; range 32–84 years) in the chronic phase. BMI was only backgroud characteritic which had significant association to IL-10 plasma concentration.

**Table 1:** The Correlation Between Interleukin(IL)-10 Plasma Concentration and Patient Characteristics of 141 Acute and 69 Chronic Phase Stroke Patients.

	Variable	Acute phase	Sig.	Chronic phase	Sig.
Characteristics	Age, y	60.5±10.6	ns.	60.3±11.3	ns.
	Males, n (%)	97 (68.8)	ns.	49 (71.0)	ns.
	Body mass index, kg/m^2^	28.0±4.5	0.008	27.4±4.1	ns.
	Body surface area, m^2^	2.0±0.2	ns.	1.9±0.2	ns.
	Caucasian race, n (%)	141 (100.0)	ns.	69 (100.0)	ns.
	Hypertension, n (%)	80 (56.7)	ns.	36 (52.2)	ns.
	Hyperlipidemia, n (%)	56 (39.7)	ns.	28 (19.9)	ns.
	Diabetes, n (%)	19 (13.5)	ns.	2 (2.9)	ns.
	Smokers, n (%)	38 (27.0)	ns.	18 (26.1)	ns.
	Stroke, n (%)	103 (73.0)	ns.	48 (69.6)	ns.
	Transient ischemic attack, n (%)	38 (27.0)	ns.	21 (30.4)	ns.
Medication during hospitalization	Aspirin, n (%)	45 (31.9)	ns.	24 (34.8)	ns.
	Warfarin, n (%)	9 (6.4)	ns.	5 (7.2)	ns.
	Clopidogrel, n (%)	3 (2.1)	0.014	1 (1.4)	ns.
	Dipyridamole, n (%)	5 (3.5)	ns.	2 (2.9)	ns.
	Statin, n (%)	40 (28.4)	ns.	18 (26.1)	ns.
IL-10 sample collection	Sample retrieval after hospitalization, days	3±2	ns.	102±13	ns.
Stroke/TIA etiology	Cardioembolic risk source (≥1)	39 (28%)	0.006	11 (16%)	ns.
	Large-artery atherosclerosis	24 (17%)	ns.	5 (7%)	ns.
	Cardioembolic source combined with large-artery atherosclerosis	9 (6%)	ns.	5 (7%)	ns.
	Small-vessel occlusion	11 (8%)	ns.	4 (6%)	ns.
	Cryptogenic	78 (55%)	0.034	44 (64%)	ns.

Sig. = significance; ns. = no significance at level p<0.05

**Fig 1:**
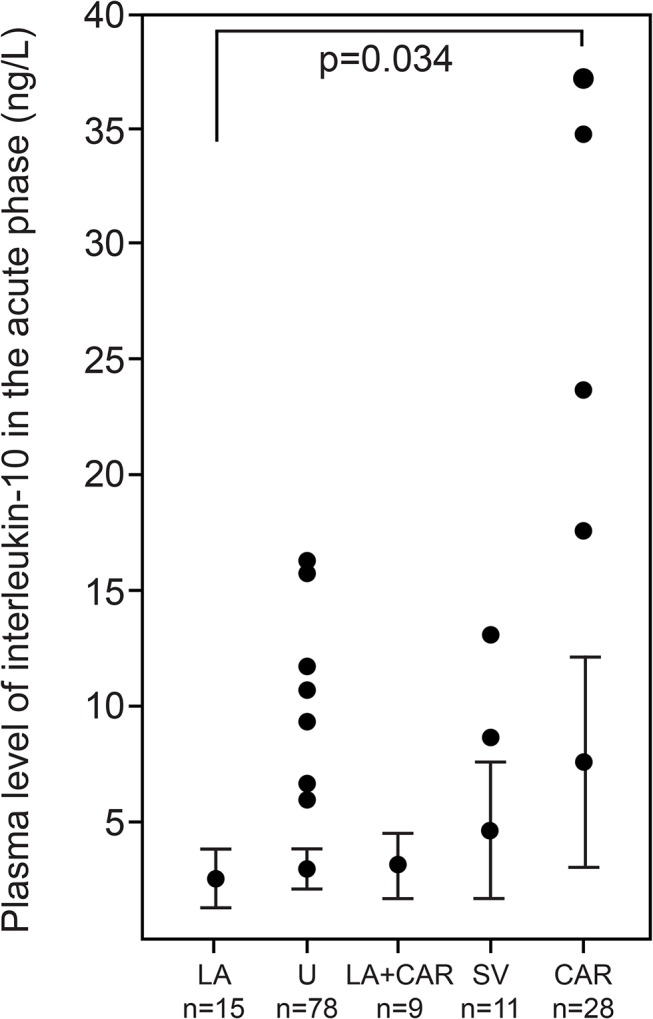
Acute Phase Interleukin(IL)-10 Plasma Concentration (ng/L) Presented with 95% Confidence Intervals in Different Stroke/Transient Ischemic Attack (TIA) Etiologies. IL-10 concentration in 28 patients (20%) with cardioembolic risk sources for stroke/TIA (CAR) had a mean IL-10 plasma concentration of 6.5±9.9 ng/L. Stroke/TIA with a large-artery atherosclerosis etiology (LA) was found in 15 patients (11%), with a mean IL-10 plasma concentration of 2.2±1.8 ng/L. Nine patients (6%) were characterized with both cardiogenic and atherosclerotic etiologies (LA+CAR), and had a mean IL-10 concentration of 2.7±1.8 ng/L. Small-vessel occlusion (SV) was found in 11 patients (8%) with a mean IL-10 plasma concentration of 4.0±3.8 ng/L; in the remaining 78 patients (55%) with undetermined etiology (U), the IL-10 plasma concentration was 2.6±3.2 ng/L. IL-10 concentration in stroke/TIA patients with major risk sources for cardioembolism was significantly higher (p = 0.034) than in patients with large-artery carotid/vertebral atherosclerosis and without risk sources for cardioembolism.

The mean IL-10 plasma concentration was 3.4±5.3 ng/L (range: 0.3–37.2 ng/L) in the acute phase and 2.5±2.6 ng/L (range 0.3–15.5 ng/L) in the chronic phase (ns). Neither acute nor chronic phase IL-10 plasma concentrations were associated with prior or recent (<6 months) ischemic MI, vertebral/carotid stenosis, given intravenous thrombolytic treatment, or infarction volume in cerebral tissue. Plasma IL-10 concentration in the acute phase was significantly higher (p<0.034) in patients with cardioembolic risk source/sources (≥1) compared to those with atherosclerotic etiology (Fig 1), the significance remained also after the IL-10 plasma concentration was adjusted for BMI. Between other etiologies, no significant differences were observed.

### IL-10 Concentration in Patients with Cardioembolic Stroke/TIA

A statistically significant association was observed between IL-10 plasma concentration and the number of detected EAE high risk sources (p = 0.023). The association between IL-10 concentration and the number of EAE high risk sources remained significant after excluding the single case with three risk factors (p = 0.036), and also after adjustment for body mass index (BMI).

**Fig 2:**
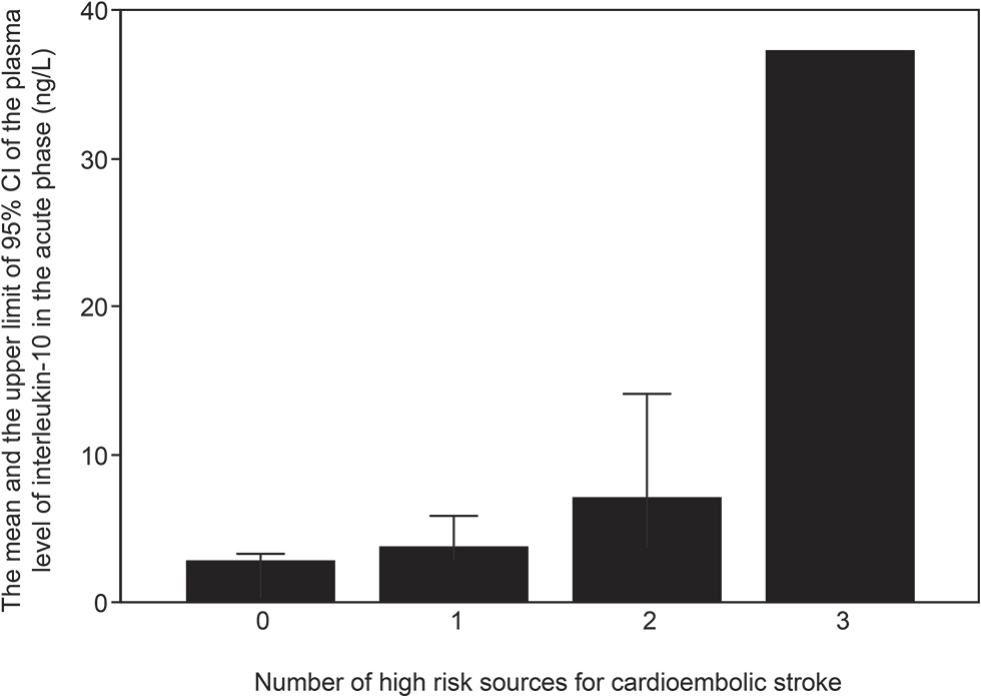
Acute Phase Interleukin (IL)-10 Plasma Concentration (ng/L) in 141 Patients with Cardioembolic Stroke According to the Number of High Risk Source Findings Presented by the European Association of Echocardiography (EAE). In patients with no EAE high risk factors, the mean IL-10 concentration was 2.7±3.1 ng/L (n = 104; range: 0.3–16.3 ng/L); with one risk factor it was 3.7±5.5 ng/L (n = 26; range: 0.3–23.6 ng/L), with two risk factors 7.0±10.0 ng/L (n = 10; range: 1.29–34.8 ng/L) and with three risk factors the IL-10 measurement was 37.2 ng/L (n = 1).

In patients with no risk factors, IL-10 concentration in the acute phase was 2.7±3.1 ng/L (n = 104; range: 0.3–16.3 ng/L), with one risk factor 3.7±5.5 ng/L (n = 26; range: 0.3–23.6 ng/L), with two risk factors 7.0±10.0 ng/L (n = 10; range: 1.29–34.8 ng/L) and with three risk factors 37.2 ng/L (n = 1) (Fig 2). No statistical significance was found between IL-10 and the number of EAE high risk sources in the chronic phase.

IL-10 levels in patients with or without each single EAE high risk sources are presented in Table 2. Mean level of IL-10 was high when previous MI, left ventricular aneurysm, intracardiac thrombus or aortic arch atheromatous plaque was present. However, the number of patients was small and several patients had multiple high risk sources. After Bonferroni correction, none of the individual risk sources correlated significantly with IL-10 concentration. Previously known AF was an exclusion criteria of the study, but patients who were found to have paroxysmal AF in 24-h Holter recording during hospital stay (n = 20) were not excluded. There was no significant difference in IL-10 plasma concentrations between patients with or without AF.

**Table 2:** Interleukin-10 Plasma Concentration in Risk Source Groups Defined by the European Association of Echocardiography (EAE).

	At hospitalization						After 3 months					
EAE high risk sources	Present		Absent		Significance		Present		Absent		Significance	
	N (%)	Mean ± SD (ng/L)	N (%)	Mean ± SD (ng/L)	Unadjusted	Bonferroni Adjusted	N (%)	Mean ± SD (ng/L)	N (%)	Mean ± SD (ng/L)	Unadjusted	Bonferroni Adjusted
Atrial fibrillation	20 (14.2)	3.0±2.6	121 (85.8)	3.5±5.6	ns.	ns.	9 (13.0)	1.5±1.2	60 (87.0)	2.7±2.7	ns.	ns.
Previous myocardial infarction	13 (9.2)	7.8±10.7	128 (90.7)	3.0±4.3	ns.	ns.	6 (8.7)	2.5±2.1	63 (91.3)	2.5±2.7	ns.	ns.
Recent myocardial infarction (left ventricular aneurysm)	1 (0.7)	37.2	140 (99.3)	3.2±4.5	n/a	n/a	0 (0.0)	-	69 (100.0)	2.5±2.6	n/a	n/a
Cardiomyopathies	0 (0.0)	-	141 (100.0)	3.4±5.3	n/a	n/a	0 (0.0)	-	69 (100.0)	2.5±2.6	n/a	n/a
Intracardiac thrombus	6 (4.3)	13.7± 17.4	135 (95.7)	3.0±3.6	ns.	ns.	0 (0.0)	-	69 (100.0)	2.5±2.6	n/a	n/a
Intracardiac tumours	2 (1.4)	3.0±2.5	139 (98.6)	3.5±5.3	n/a	n/a	0 (0.0)	-	69 (100.0)	2.5±2.6	n/a	n/a
Fibroelastoma	1 (0.7)	0.6±0.0	140 (99.3)	3.5±5.3	n/a	n/a	0 (0.0)	-	69 (100.0)	2.5±2.6	n/a	n/a
Marantic vegetations	0 (0.0)	-	141 (100.0)	3.4±5.3	n/a	n/a	0 (0.0)	-	69 (100.0)	2.5±2.6	n/a	n/a
Rheumatic valve disease (mitral stenosis)	0 (0.0)	-	141 (100.0)	3.4±5.3	n/a	n/a	0 (0.0)	-	69 (100)	2.5±2.6	n/a	n/a
Aortic arch atheromatous plaques	6 (4.3)	9.8± 13.6	135 (95.7)	3.2±4.5	0.023	ns.	1 (1.4)	0.8±0.0	68 (98.6)	2.5±2.6	ns.	ns.
Endocarditis	0 (0.0)	-	141 (100.0)	3.4±5.3	n/a	n/a	0 (0.0)	-	69 (100)	2.5±2.6	n/a	n/a
Mechanical valve prosthesis	0 (0.0)	-	141 (100.0)	3.4±5.3	n/a	n/a	0 (0.0)	-	69 (100)	2.5±2.6	n/a	n/a

SD = standard deviation; ns. = no significance at level p<0.05 or <0.003 for unadjusted and adjusted significances, respectively; n/a = not applicable.

## Discussion

The main finding of the current study was that plasma IL-10 concentration in stroke patients with major risk sources for cardioembolism (highly probable cardiogenic etiology) was significantly higher than in patients with large-artery carotid/vertebral atherosclerosis and without major risk sources for cardioembolism (highly probable non-cardiogenic etiology) (Fig 1). Moreover, plasma IL-10 level was associated with the number of cardioembolic risk sources; the higher the number, the higher the measured acute phase IL-10 concentration (Table 3). The association of IL-10 concentrations could be seen only at the acute phase of stroke; three months later there was no difference in IL-10 levels between the categories. This suggests that IL-10 secretion is related to acute cardiogenic thrombus, either to its formation, resolution or to the acute consequences of its embolism, and might have potential to improve the differential diagnostics of stroke/TIA etiology.

**Table 3:** Interleukin-10 Plasma Concentration in Different Stroke Patient Categories Defined by the Total Number of the European Association of Echocardiography High Risk Sources.

	At hospitalization		After three months	
Number of high risk sources	N (%)	Mean±SD (ng/L)	N (%)	Mean±SD (ng/L)
0	104 (73.8)	2.7±3.1	54 (78.3)	2.7±2.8
1	26 (18.4)	3.7±5.5	14 (20.3)	2.0±1.7
2	10 (7.1)	7.0±10.0	1 (1.4)	1.7±1.2
3	1 (0.7)	37.2±0.0	0 (0.0)	0.0±0.0

SD = standard deviation.

Previously, Emsley et al. have reported that patients with acute ischemic stroke and significant large artery atherosclerosis have significantly lower concentrations of plasma IL-10 than stroke patients without significant atherosclerosis [19]. Our results are in line with their observation. However, in the study of Emsley [19] major sources of cardioembolism, and their association with IL-10 were not evaluated. To the best of our knowledge, the association between the presence of cardioembolic risk sources and IL-10 levels has neither been reported elsewhere before. In our study, the patients were thoroughly investigated by cardiac imaging, and patients with ≥ 1 detected visible cardioembolic sources (left chamber aneurysm, thrombus detected by TTE, >4 mm aortic arch atheromatous plaque and previous myocardial infarction with abnormal left ventricle wall motion) had the highest plasma IL-10 concentrations.

AF is the most common etiology for cardioembolic stroke [4,5]. Our study was aimed to assess patients with unknown or suspected (but not obvious) cardiogenic stroke etiology. Therefore, previously known atrial fibrillation was an exclusion criteria. However, patients who were diagnosed to have paroxysmal AF (PAF) in 24-h Holter recording during hospital stay (n = 20) were not excluded. This subgroup of patients was relatively small (14.2% of the whole population) and selected, and does not represent the whole spectrum of AF. Thereby, strong conclusions of the association between AF and IL-10 plasma concentration cannot be drawn. Interestingly, we found mean IL-10 concentration low in this PAF population.

In an animal model, both intraventricularly and systemically administered IL-10 have been shown to reduce cerebral infarction volume [13], and IL-10 has been suggested to be neuroprotective [13–15, 20]. High levels of serum IL-10 have been suggested to facilitate the selection of ischemic stroke patients with salvageable brain tissue for systemic thrombolysis [14]. However, no association with the IL-10 serum concentration and the cerebral infarction volume was found in our study.

IL-10, an anti-inflammatory cytokine produced primarily by T-cells and monocytes, is suggested to play a role in vascular protection, although the exact mechanisms remain unclear. IL-10 has been shown to reduce atherogenesis via inhibition of the LDL/Ox-LDL dependent monocyte-endothelial interaction [21–23]. In addition, low production of IL-10 has been associated with several cardiovascular risk factors, such as metabolic syndrome, insulin resistance and type 2 diabetes mellitus [24,25], and overweight-related sleep apnea [26]. Decreased IL-10 levels are found at the onset of acute coronary syndrome and increased concentrations have been associated with improved prognosis [27–32]. Interestingly, IL-10 knockout mice have been reported to develop vascular and cardiac dysfunction [33]. In addition, epidemiological data indicate that individuals with elevated IL-10 plasma levels have a reduced risk of stroke [34], and that subjects with a history of stroke have lower levels of IL-10 than those without previous stroke [24].

As the increased level of IL-10 seems to be vascular- and cardioprotective according to the previous literature, IL-10 is not likely to be the predisposing factor for the stroke in our cardioembolic subgroup, either. More likely, the acutely elevated IL-10 might play a role in the autoregulatory or protective feedback mechanism during thrombus generation. This presumption is supported by several previous findings. Earlier, Downing et al. (1998) have demonstrated in an animal model that emerging high quantities of IL-10 secreted by monocytes and neutrophils consequently to mechanical stasis-induced venous thrombosis regulate the resultant vein wall inflammatory response [10]. Moreover, IL-10 has been shown to reduce procoagulant and proaggregatory activity in various *in vitro* and *in vivo* models [22,30,35–40]. The main limitation of this study was the relatively small number of patients with more than two EAE risk sources for cardioembolic stroke. The study population decreased even further over the three month follow-up due to low attendance to follow-up visits. The treatment of the patients was not standardized, but was tailored individually according to the current guidelines. Therefore, there is a possibility that antithrombotic treatment or other medication might have interfered with the results. Eleven patients (7.8%) were treated with recombinant tissue plasminogen activator (r-tPA). The IL-10 levels of this small group did not differ from the rest of the population, suggesting that plasma IL-10 concentration was not affected by the administration of r-tPA.

In conclusion, we found significant difference in IL-10 concentration between patients with cardioembolic sources and large-artery atherosclerosis in acute-phase of stroke. IL-10 plasma concentration was strongly associated with the number of cardioembolic high risk sources. The results merit further studies in larger stroke populations, also including AF patient groups. According to our preliminary results, IL-10 measurement may potentially improve the diagnostics of stroke etiology.

## References

[1] Lo EH, Dalkara T, Moskowitz MA. Mechanisms, challenges and opportunities in stroke. Nature Reviews. Neuroscience. 2003;4:399–415. 12728267 10.1038/nrn1106

[2] Murray CJL, Lopez AD. Mortality by cause for eight regions of the world: Global burden of disease study. Lancet. 1997;349(9061):1269–1276. 9142060 10.1016/S0140-6736(96)07493-4

[3] Yip P, Jeng J, Lee T, Chang Y, Huang Z, Ng ZS, et al Subtypes of ischemic stroke. A hospital-based stroke registry in Taiwan (SCAN-IV). Stroke. 1997;28(12):2507–2512. 9412641 10.1161/01.str.28.12.2507

[4] Ferro JM. Cardioembolic stroke: An update. Lancet Neurology. 2003;2(3):177–188. 12849239 10.1016/s1474-4422(03)00324-7

[5] D'Olhaberriague L, Hernández-Vidal A, Molina L, Soler-Singla L, Marrugat J, Pons S, et al A prospective study of atrial fibrillation and stroke. Stroke. 1989;20(12):1648–1652. 2688196 10.1161/01.str.20.12.1648

[6] Bang OY, Lee PH, Joo SY, Lee JS, Joo IS, Huh K. Frequency and mechanisms of stroke recurrence after cryptogenic stroke. Annals of Neurology. 2003;54(2):227–234. 12891675 10.1002/ana.10644

[7] Taina M, Vanninen R, Hedman M, Jäkälä P, Kärkkäinen S, Tapiola T, et al Left atrial appendage volume increased in more than half of patients with cryptogenic stroke. PLoS ONE. 2013;8(11):e79519 10.1371/journal.pone.0079519 24223960 PMC3817123

[8] Sipola P, Hedman M, Onatsu J, Turpeinen A, Halinen M, Jäkälä P, et al Computed tomography and echocardiography together reveal more high-risk findings than echocardiography alone in the diagnostics of stroke etiology. Cerebrovascular Diseases. 2013;35(6):521–530. 10.1159/000350734 23817231

[9] Adams HJ, Bendixen BH, Kappelle LJ, Biller J, Love BB, Gordon DL, et al Classification of subtype of acute ischemic stroke. Definitions for use in a multicenter clinical trial. TOAST. trial of org 10172 in acute stroke treatment. Stroke. 1993;24(1):35–41. 7678184 10.1161/01.str.24.1.35

[10] Downing L, Strieter J, Kadell RM, Wilke AM, Austin CA, Hare JC, et al IL-10 regulates thrombus-induced vein wall inflammation and thrombosis. Journal of Immunology. 1998;161(3):1471–1476. 9686613

[11] Tarkowski E, Rosengren L, Blomstrand C, Wikkelsö C, Jensen C, Ekholm S, et al Intrathecal release of pro- and anti-inflammatory cytokines during stroke. Clinical and Experimental Immunology. 1997;110(3):492–499. 9409656 10.1046/j.1365-2249.1997.4621483.x PMC1904815

[12] Lakhan SE, Kirchgessner A, Hofer M. Inflammatory mechanisms in ischemic stroke: Therapeutic approaches. Journal of Translational Medicine. 2009;7:97 10.1186/1479-5876-7-97 19919699 PMC2780998

[13] Spera PA, Ellison JA, Feuerstein GZ, Barone FC. IL-10 reduces rat brain injury following focal stroke. Neuroscience Letter. 1998;251(3):189–192. 9726375 10.1016/s0304-3940(98)00537-0

[14] Rodríguez-Yáñez M, Sobrino T, Arias S, Vázquez-Herrero F, Brea D, Blanco M, et al Early biomarkers of clinical-diffusion mismatch in acute ischemic stroke. Stroke. 2011;42(10):2813–2818. 10.1161/STROKEAHA.111.614503 21836082

[15] Strle K, Zhou JH, Shen WH, Broussard SR, Johnson RW, Freund GG, et al Interleukin-10 in the brain. Critical Reviews of Immunology. 2001;21(5):427–449. 11942558

[16] Pelidou S, Kostulas N, Matusevicius D, Kivisäkk P, Kostulas V, Link H. High levels of IL-10 secreting cells are present in blood in cerebrovascular diseases. European Journal of Neurology. 1999;6(4):437–442. 10362896 10.1046/j.1468-1331.1999.640437.x

[17] Pepi M, Evangelista A, Nihoyannopoulos P, Flachskampf FA, Athanassopoulos G, Colonna P, et al (2010) Recommendations for echocardiography use in the diagnosis and management of cardiac sources of embolism: European Association of Echocardiography (EAE) (a registered branch of the ESC). Eur J Echocardiogr 11: 461–476. 10.1093/ejechocard/jeq045 20702884

[18] Graham TJ, Jarmakani J, Atwood G, Canent RJ. Right ventricular volume determinations in children. normal values and observations with volume or pressure overload. Circulation. 1973;47:144–153. 4686591 10.1161/01.cir.47.1.144

[19] Emsley H, Smith C, Gavin C, Georgiou RF, Vail A, Barberan EM, et al Clinical outcome following acute ischaemic stroke relates to both activation and autoregulatory inhibition of cytokine production. BMC Neurology. 2007;7(1):5. 17328808 10.1186/1471-2377-7-5 PMC1810309

[20] Pelidou SH, Kostulas N, Mutusevicius D, Kivisäkk P, Kostulas V, Link H. High levels of IL-10 secreting cells are present in blood in cerebrovascular diseases. European Journal of Neurology. 1999;6(4):437–442. 10362896 10.1046/j.1468-1331.1999.640437.x

[21] Caligiuri G, Rudling M, Ollivier V, Jacob MP, Michel JB, Hansson GK, et al Interleukin-10 deficiency increases atherosclerosis, thrombosis, and low-density lipoproteins in apolipoprotein E knockout mice. Molecular Medicine. 2003;9(1–2):10–17. 12765335 PMC1430379

[22] Mallat Z, Besnard S, Duriez M, Deleuze V, Emmanuel F, Bureau MF, et al Protective role of interleukin-10 in atherosclerosis. Circulation Research. 1999;85(8):e17–e24. 10521249 10.1161/01.res.85.8.e17

[23] Pinderski OL, Hedrick C, Olvera T, Hagenbaugh A, Territo M, Berliner JA, et al Interleukin-10 blocks atherosclerotic events in vitro and in vivo. Arteriosclerosis, Thrombosis, and Vascular Biology. 1999;19(12):2847–2853. 10591660 10.1161/01.atv.19.12.2847

[24] van Exel E, Gussekloo J, de Craen A, Bootsma-van der Wiel A, Frölich M, Westendorp R. Inflammation and stroke: The leiden 85-plus study. Stroke. 2002;33(4):1135–1138. 11935072 10.1161/01.str.0000014206.05597.9e

[25] Aroor A, McKarns S, Demarco V, Jia G, Sowers J. Maladaptive immune and inflammatory pathways lead to cardiovascular insulin resistance. Metabolism. 2013;62(11):1543–1552. 10.1016/j.metabol.2013.07.001 23932846 PMC3809332

[26] Sahlman J, Miettinen K, Peuhkurinen K, Seppä J, Peltonen M, Herder C, et al The activation of the inflammatory cytokines in overweight patients with mild obstructive sleep apnoea. Journal of Sleep Research. 2010;19(2):341–348. 10.1111/j.1365-2869.2009.00787.x 20040038

[27] Anguera I, Miranda-Guardiola F, Bosch X, Filella X, Sitges M, Marín JL, et al Elevation of serum levels of the anti-inflammatory cytokine interleukin-10 and decreased risk of coronary events in patients with unstable angina. American Heart Journal. 2002;144(5):811–817. 12422149 10.1067/mhj.2002.124831

[28] Kato K, Oguri M, Hibino T, Yajima K, Matsuo H, Segawa T, et al Genetic factors for lone atrial fibrillation. International Journal of Molecular Medicine. 2007;19(6):933–939. 17487426

[29] Heeschen C, Dimmeler S, Hamm C, Fichtlscherer S, Boersma E, Simoons ML, et al Serum level of the antiinflammatory cytokine interleukin-10 is an important prognostic determinant in patients with acute coronary syndromes. Circulation. 2003;107(16):2109–2114. 12668510 10.1161/01.CIR.0000065232.57371.25

[30] Uyemura K, Demer L, Castle S, Jullien D, Berliner JA, Gately MK, et al Cross-regulatory roles of interleukin (IL)-12 and IL-10 in atherosclerosis. The Journal of Clinical Investigation. 1996;97(9):2130–2138. 8621803 10.1172/JCI118650 PMC507288

[31] Kilic T, Ural D, Ural E, Yumuk Z, Agacdiken A, Sahin T, et al Relation between proinflammatory to anti-inflammatory cytokine ratios and long-term prognosis in patients with non-ST elevation acute coronary syndrome. Heart. 2006;92(8):1041–1046. 16547209 10.1136/hrt.2005.080382 PMC1861097

[32] Leftheriotis DI, Fountoulaki KT, Flevari PG, Parissis JT, Panou FK, Andreadou KS, et al The predictive value of inflammatory and oxidative markers following the successful cardioversion of persistent lone atrial fibrillation. International Journal of Cardiology. 2009;135(3):361–369. 18640731 10.1016/j.ijcard.2008.04.012

[33] Sikka G, Miller K, Steppan J, Pandey D, Jung SM, Fraser CD 3rd, et al Interleukin 10 knockout frail mice develop cardiac and vascular dysfunction with increased age. Experimental Gerontology. 2013;48(2):128–135. 10.1016/j.exger.2012.11.001 23159957 PMC3744178

[34] Xie G, Myint P, Zaman M, Li Y, Zhao L, Shi P, et al Relationship of serum interleukin-10 and its genetic variations with ischemic stroke in a chinese general population. PLoS One. 2013;8(9):e74126 10.1371/journal.pone.0074126 24040186 PMC3770660

[35] Jungi T, Brcic M, Eperon S, Albrecht S. Down-regulation of fibrinogen biosynthesis by IL-4, IL-10 and IL-13. Thrombosis Research. 1994;76(5):463–474. 7900094 10.1016/0049-3848(95)90178-i

[36] Vasse M, Paysant J, Soria J, Mirshahi S, Vannier J, Soria C. Down-regulation of fibrinogen biosynthesis by IL-4, IL-10 and IL-13. British Journal Haematology. 1996;96(5):955–961. 10.1046/j.1365-2141.1996.d01-1731.x 8703833

[37] Pajkrt D, van der Poll T, Levi M, Cutler DL, Affrime MB, van den Ende A, et al Interleukin-10 inhibits activation of coagulation and fibrinolysis during human endotoxemia. Blood. 1997;89(8):2701–2705. 9108387

[38] Gudbrandsdottir S, Hasselbalch H, Nielsen C. Activated platelets enhance IL-10 secretion and reduce TNF-a secretion by monocytes. The Journal of Immunology. 2013;191(8):4059–4067. 10.4049/jimmunol.1201103 24048901

[39] Hagihara M, Higuchi A, Tamura N, Ueda Y, Hirabayashi K, Ikeda Y, et al Platelets, after exposure to a high shear stress, induce IL-10-producing, mature dendritic cells in vitro. Journal of Immunology. 2004;172(9):5297–5303. 15100268 10.4049/jimmunol.172.9.5297

[40] Osmancik P, Paulu P, Tousek P, Kocka V, Widimsky P. High leukocyte count and interleukin 10 predict high on-treatment-platelet-reactivity in patients treated with clopidogrel. Journal of Thrombosis and Thrombolysis. 2012;33(4):349–354. 22189756 10.1007/s11239-011-0659-5

